# Recurrent cholangitis due to an intrahepatic calculus caused by migrated coil for vascular embolization: A case report

**DOI:** 10.1097/MD.0000000000041038

**Published:** 2024-12-20

**Authors:** Naotake Funamizu, Kyosei Sogabe, Mio Uraoka, Yuki Numata, Mitsuhito Koizumi, Chihiro Ito, Yoshitomo Ueno, Yoshio Ikeda, Yuzo Umeda

**Affiliations:** a Department of Hepatobiliary Pancreatic Surgery, Ehime University Graduate School of Medicine, Toon City, Ehime, Japan; b Department of Gastroenterology and Metabology, Ehime University Graduate School of Medicine, Toon City, Ehime, Japan.

**Keywords:** case report, coil, Intervention radiology, intrahepatic calculi, recurrent cholangitis

## Abstract

**Rationale:**

Pseudoaneurysm is a potential postoperative complication in hepatobiliary and pancreatic surgery, with catheter-based interventions being the first-line treatment. This study reviews the literature on potential secondary complications following arterial embolization. Additionally, we report a case in which a dislodged embolization coil acted as a nidus for bile duct stone formation, leading to recurrent cholangitis. This report aims to raise awareness among clinicians regarding such clinical scenarios.

**Patient concerns:**

In the current report, we discuss the case of a 43-year-old male patient, who had undergone coil embolization due to a hepatic artery pseudoaneurysm after biliary reconstruction because of bile duct injury during the laparoscopic cholecystectomy, was admitted to our hospital for repeated cholangitis.

**Diagnoses:**

Imaging modalities confirmed that the previously embolized coil had migrated into the bile duct, which was identified as the cause.

**Interventions:**

A double-balloon endoscopy revealed stones with a migrated coil as its nucleus.

**Outcomes:**

The endoscopic stone removal was completed.

**Lessons:**

We encountered a case in which an arterial embolization coil used for the treatment of a pseudoaneurysm migrated into the bile duct, acting as a nidus for stone formation and resulting in recurrent cholangitis. In patients with a history of intrahepatic coil embolization, it is essential to first confirm the location of the coil within the vasculature and then investigate the underlying cause of stone formation. It is important to consider coil migration as a differential diagnosis in cases of bile duct stones following hepatic artery embolization with coils.

## 1. Introduction

One of the fatal postoperative complications (POCs) is intra-abdominal hemorrhage caused by the rupture of a pseudoaneurysm in hepato–biliary–pancreatic surgery.^[1]^ Reported contributing factors include arterial stump bleeding, arterial wall damage caused by surgical manipulation, and exposure to digestive fluids due to anastomotic failure.^[2]^ Generally, interventional radiology (IVR) is considered the first-line treatment choice. Conversely, the risk of developing cholangitis is caused by biliary-enteric anastomotic stricture and bile reflux due to increased intestinal pressure after hepato–biliary–pancreatic surgery. However, bile duct stones caused by coil migration are an exceedingly rare complication. Herein, we report a case of recurrent cholangitis 2 years after coil embolization for an arterial pseudoaneurysm. The recurrence was associated with the coil inadvertently migrating into the bile duct, forming intrahepatic calculi centered around the coil.

## 2. Case report

**Figure 1.**
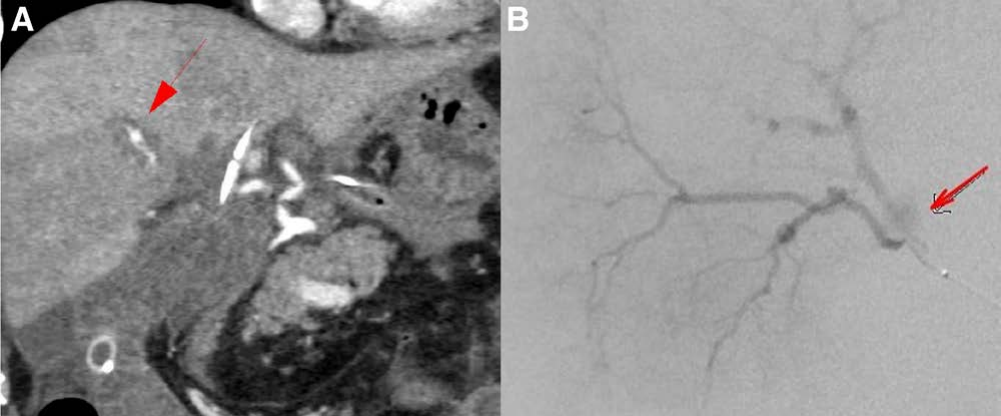
Biliary bleeding was observed following biliary reconstruction surgery. (A) Prompting a contrast-enhanced CT scan which revealed an intrahepatic aneurysm(➡). (B) Emergency IVR was performed. Angiography revealed a ruptured aneurysm in the A8 hepatic artery (➡), which was successfully coiled for hemostasis 2 years ago. CT = computed tomography, IVR = interventionl radiology.

A 44-year-old male patient with repeated cholangitis and jaundice was referred from a previous hospital for further assessment. The patient reported a history of a choledochoenterostomy at our hospital 2 years ago, because he experienced a bile duct injury during laparoscopic cholecystectomy at another hospital causing bile leakage, which was subsequently managed with choledochoenterostomy. The patient presented with bleeding from the tube placed in the bile duct and a sudden onset of severe abdominal pain on postoperative day 6 after biliary reconstruction surgery. Emergent CT revealed a ruptured aneurysm of the hepatic artery (A8; Fig. 1A). Thus, the patient underwent treatment with IVR (Fig. 1B). Thereafter, the patient recovered smoothly and was regularly followed up at the previous hospital.

**Figure 2.**
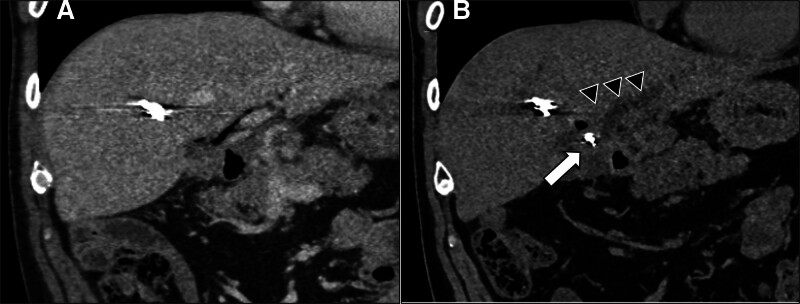
Comparison between the CT scan performed after coil embolization of the aneurysm 2 years ago and the CT scan upon admission. (A) CT scan performed after coil embolization of the hepatic artery (A8) aneurysm following biliary reconstruction surgery 2 years ago. (B) In the CT scan upon admission, partial migration of the coil to the hepatic hilum (➡) was observed, along with dilation of the intrahepatic bile ducts (▲). CT = computed tomography.

During this hospitalization, an enhanced CT revealed that a portion of the coil, which was used for embolization of the A8 hepatic artery, had migrated into the hepatic hilum (Fig. 2A). Furthermore, intrahepatic bile duct dilation was observed (Fig. 2B).

**Figure 3.**
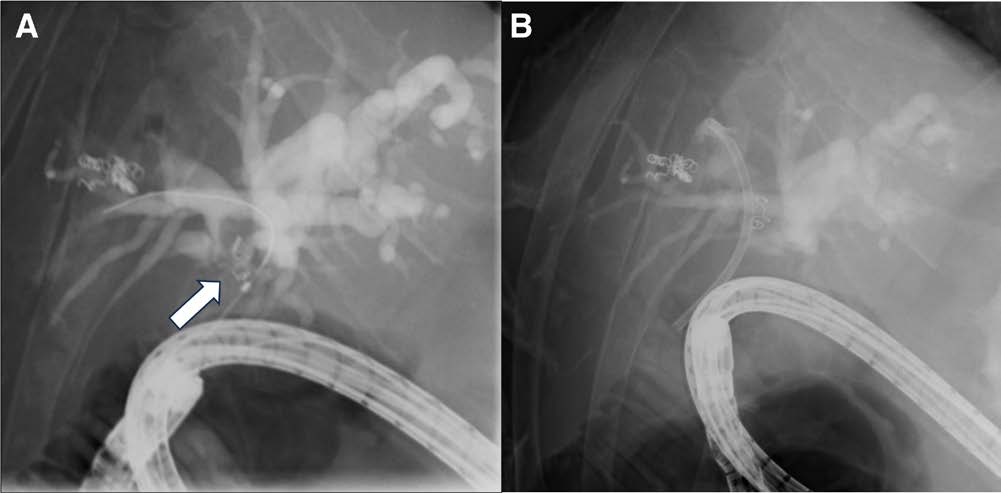
Double-balloon endoscopy findings. (A) A translucency with a coil is observed at the choledochojejunal anastomosis, with a size measuring 15 mm. (B) Due to challenging stone extraction, a plastic stent was placed.

**Figure 4.**
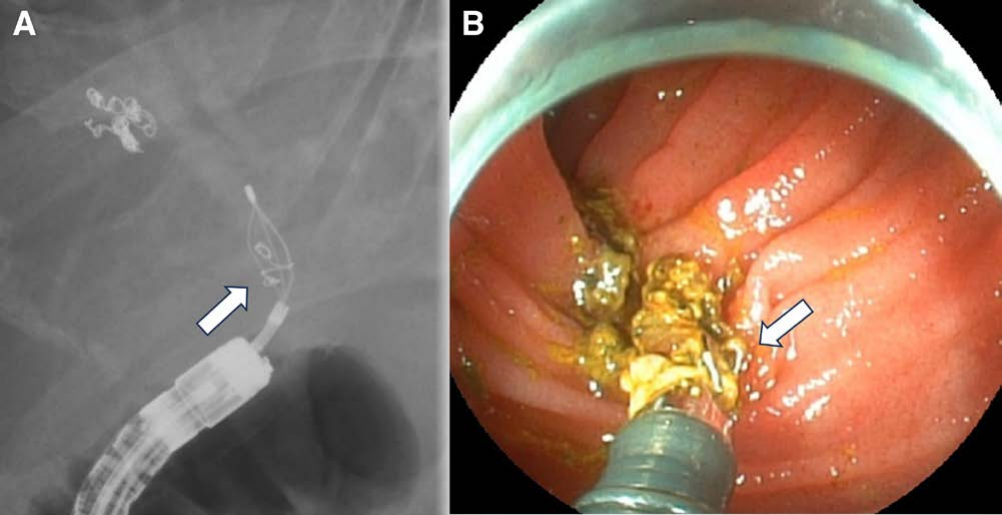
Second ERC was performed. (A) The stones were fragmented using a lithotripter grasper. (B) The stones containing a coil were successfully removed.

Based on those results, a double-balloon endoscopy (DBE) was conducted. DBE revealed that stones, centered around the coil, were determined at the anastomotic site (Fig. 3A). Concurrently, stone extraction was not possible, and a plastic stent was placed (Fig. 3B). The anastomosis was dilated with an 8-mm balloon during the second DBE, followed by stone fragmentation using a crusher basket (Fig. 4A), causing successful stone removal. The coil was confirmed to be included when removing the stones with basket forceps (Fig. 4B).

## 3. Discussion

The incidence of coil migration after arterial embolization is 0.8%, with a higher prevalence of being observed as ulcers in the gastrointestinal tract and rarely within the biliary ducts.^[3]^ Various reports have documented foreign bodies, such as surgical clips,^[4]^ fish bones,^[5]^ and others,^[6]^ that have migrated into the bile ducts, excluding vascular embolization coils.

Conversely, the reported incidence of hepatic artery pseudoaneurysm after laparoscopic cholecystectomy is 0.25%.^[7]^ Late intra-abdominal bleeding after laparoscopic cholecystectomy is most predominantly attributed to a pseudoaneurysm of the right hepatic artery in 60% of patients when analyzed by artery, followed by the common hepatic artery in 30%, the cystic artery in 10%, and occasionally the gastroduodenal artery.^[8]^ These data indicated that it is rare for cases of coil migration into the bile duct after coiling of pseudoaneurysms following laparoscopic cholecystectomy.

Therefore, only 11 cases of vascular embolization coil migration in the bile duct have been reported as per our search on PubMed.^[9–19]^ Table 1 compiles and summarizes these reports, including this case, resulting in 12 cases. Many of the reported cases so far have demonstrated pseudoaneurysm formation in the right hepatic artery associated with POCs after cholecystectomy, liver transplantation, traffic injury, and percutaneous transhepatic biliary drainage. Furthermore, the duration from IVR to coil migration into the bile duct ranged from 2 months to over 10 years, with no consistent pattern. Meanwhile, coil removal was successfully performed in all cases. Additionally, many cases were removed endoscopically. However, reports using balloon-assisted endoscopy, such as our case, were limited, since cases after biliary reconstruction were typically addressed through either reoperation or percutaneous approaches.

**Table 1:** Summary of case reports for coil migration into the bile duct following coil treatment for pseudo aneurysms on Pubmed.

No.	Year	Author	Sex	Age	Diagnosis before aneurysm	Etiology of aneurysms	The artery of aneurysm	Duration for migration (mo)	Treatment	Approach methods
1	2002	Ozkan	M	58	AC	C	RHA	24	Reconstruction	Laparotomy
2	2007	Turaga	M	65	AC, CBD stone	C, Choledochotomy	RHA	12	Choledochotomy	Laparotomy
3	2007	Van Steenbergen	N	72	Cirrhosis	LT	RHA	60	Lithotripsy	Endoscopy
4	2009	Zaafouri	M	55	Cholangitis, CBD stone	C, Choledochotomy	GDA	20	Lithotripsy	Endoscopy
5	2011	Kao	F	65	AC, CBD stone	C, Choledochotomy	RHA	96	Lithotripsy	Endoscopy
6	2012	AlGhamdi	F	55	Cirrhosis	LT	RHA	3	Lithotripsy	Endoscopy
7	2015	Raashed	M	37	Cholecystolithiasis	C➡Biliary reconstruction	RHA	2	Reconstruction	Laparotomy
8	2016	Bent	F	58	AC after RYB	LC➡PTBD	RHA	28	Lithotripsy	PTBD
9	2018	Zuberi	M	55	Traffic injuries	Traffic injuries	GDA	6	Choledochoejunostomy	Laparotomy
10	2019	Taibi	F	61	AC, CBD stone	LC	RHA	3	Lithotripsy	Endoscopy
11	2023	Elsayed	F	76	CBD stone after RYB	PTBD	RHA	120	Lithotripsy	PTBD
12	2024	Funamizu	M	43	AC	LC➡Biliary reconstruction	RHA	24	Lithotripsy	BE

AC = acute cholecystitis, BE = balloon assisted endoscopy, C = cholecystectomy, CBD = common bile duct, GDA = gastroduodenal artery, LC = laparoscopic cholecystectomy, LT = liver transplantation, PTBD = percutaneous transhepatic biliary drainage, RHA = right hepatic artery, RYB = Roux-en Y bypass.

This case presented a rare condition in which a coil used for arterial aneurysm embolization migrated into the bile duct. Consequently, the change in the coil’s position, which had previously been within the artery, went unnoticed. Furthermore, due to the altered anatomical route caused by the biliary-enteric anastomosis, it is presumed that reaching the anastomotic site would not have been possible without the use of a DBE.

In Japan, balloon-assisted endoscopy is actively used to treat bile duct stones after gastrointestinal reconstruction, reporting favorable outcomes.^[20]^ Itokawa et al^[21]^ revealed that balloon-assisted endoscopy is successful in removing bile duct stones after biliary reconstruction in >90% of patients

In conclusion, this case serves as an important reminder to consider coil-induced intrahepatic calculi as one of the potential causes of repeated cholangitis, if the patient reports a history of hepatic arterial aneurysm treatment using IVR. Furthermore, balloon endoscopy may be effective for bile duct foreign body removal, even in cases after biliary reconstruction.

## Acknowledgements

The authors would like to thank Enago (www.enago.jp) for the English language review.

## Author contributions

**Conceptualization:** Naotake Funamizu.

**Data curation:** Kyosei Sogabe.

**Project administration:** Mio Uraoka, Yuki Numata, Mitsuhito Koizumi, Chihiro Ito, Yoshitomo Ueno.

**Supervision:** Yoshio Ikeda, Yuzo Umeda.

**Writing – original draft:** Naotake Funamizu.

**Writing – review & editing:** Yoshio Ikeda, Yuzo Umeda.
